# When secretion turns into excretion – the different roles of IgA

**DOI:** 10.3389/fimmu.2022.1076312

**Published:** 2022-12-21

**Authors:** Richard A. Strugnell

**Affiliations:** Department of Microbiology & Immunology, The University of Melbourne at the Peter Doherty Institute of Infection and Immunity, Melbourne, VIC, Australia

**Keywords:** pIgR, secretory IgA, inflammation, antigen excretion, infection immunity

## Abstract

IgA deficiency is the commonest immunodeficiency affecting up to 1 in 700 individuals. The effects of IgA deficiency are difficult to see in many individuals, are mild in many fewer and severe in fewer still. While monovalent IgA is found in serum, dimeric IgA is secreted through mucosal surfaces where it helps to maintain epithelial homeostasis. Studies with knockout mice have taught us that there are subtle inflammatory consequences of removing secretory IgA (sIgA), and the best explanation for these changes can be related by the loss of the ‘excretory’ immune system. The excretion of antigens is a logical process in regulating the immune system, given the long half-life of complement fixing antibodies. But the function of IgA as an immune or inflammation regulator may go beyond antigen removal.

## Introduction

The mammalian immune system is comprised of effector mechanisms and mechanisms that have evolved to dampen or truncate these fundamental and important protective processes. In the T cell response to antigens, and depending on the biochemical milieu, the activated T cells can adapt to become potent killers, or cytokine secretors that reduce the pathogen or cancer cell burden, or instead function as regulatory T cells, where the cytokines produced dampen inflammation.

Antibodies clearly play a central role in immunity, witnessed by recurrent infections of individuals with genetic B cell deficiency often caused by pathogens of ‘intermediate virulence’ such as Gram positive bacteria *Streptococcus pneumoniae* and *Staphylococcus aureus*, and more opportunistic Gram-negative bacteria including *Pseudomonas* spp. and *Haemophilus influenzae* (1). However, this enhanced level of infection sensitivity typically falls short of that observed with T cell deficiency, including infections by opportunistic pathogens such as *Pneumocystis* spp. in HIV/AIDS.

When specific antibody isotypes are absent, the effects can be even more subtle and while evolution rarely yields fully redundant processes, it took an innovative hypothesis developed by Michael Lamm and colleagues (2–4) to fully explain the role of secretory IgA (sIgA) in health and disease. Lamm et al. demonstrated that sIgA was often ‘excreted’ as an immune complex, not (as drawn in textbooks) as an antibody with ‘empty’ antigen binding sites. This excretion leads to the removal of the bound antigen from the systemic compartment and sub-epithelial tissues, by the secretory process. Subsequently it was shown that, when this ‘excretion’ was missing, chronic peri-epithelial inflammation occurred (5).

In summary, IgA can act as a monomer in serum, and can be secreted as a J-chain complexed dimer, with some potential to block infections. It also appears to have a major a role in antigen *excretion* and this review addresses the different roles of IgA and sIgA, including the so-called ‘excretory’ functions of sIgA, from a historical perspective.

## IgA production and the pIgR^-/-^ mouse

IgA like all the immunoglobulins is synthesized as two heavy chains and two light chains. In humans, there are two isotypes of IgA – IgA1 and IgA2. Recent data suggests that two these isotypes may not have identical functions (6). The alpha heavy chains are expressed from the heavy chain locus and the light chains from the common kappa and lambda loci. The monomeric IgA antibody formed within B cells can access the Fc receptor specific for alpha heavy chain (FcαR1) and these receptors may have unique immunoregulatory roles (7). IgA can adopt a different structure from other antibodies, a ‘T’ rather than ‘Y’ shape (8) the biological consequences of which are unknown; IgA also has the shortest serum half life of the major immunoglobulins, at least in mice (9). IgA is produced by two different types of B cells, so-called B-1 and B-2 B cells, and is the most abundantly produced immunoglobulin (10, 11) albeit the majority of IgA is excreted where the secretory antibody pathway is intact, and accumulates when it is absent (12).

The B-1 B cells are held as more innate-like and T-independent cells whereas the B-2 cells receive T cell help, undergo more typical selection and produce higher affinity antibody (13). At least some of the IgA antibody produced by B-1 cells is seen as ‘polyreactive’ or ‘polyspecific’ insofar as monoclonal antibodies derived from these B-1 cells have unusual CDR features with respect to charge and length that explain their binding to more than one antigen (14–17), albeit often with much reduced affinity. In the absence of conventional B-2 cells, IgA may still be present (18) suggesting there are quite different developmental lineages for B-1 and B-2 cells. These pathways have been recently reviewed (19). The antigenic specificity of sIgA is strongly influenced by the microbiome present, though the route through which the immune system engages with the microbiome influences the ‘spreading’ of antibody class and specificity (20). In germ free systems, mucosal challenge by a defined microbiome is met with a restricted, oligoclonal sIgA repertoire compared with animals provided the same organisms intravenously, where the antibodies include IgG of an increased number of specificities.

In the absence of the joining or ‘J-chain’, antibody of the IgA class is destined to remain in the circulation (21). Synthesis by the IgA producing B cells of J-chain leads to a predominantly divalent antibody carrying the J-chain that can be secreted across the mucosal epithelia by the polyimmunoglobulin receptor (pIgR). The pIgR, a member of the immunoglobulin superfamily, is expressed on the basolateral surfaces of mucosal epithelia meaning that plasma j-chain containing IgA antibody can be secreted from any of the mucosal surfaces. Where the antibody secreting B cells or plasma cells are mucosal tissue resident, the levels of sIgA may be higher but, generally, the distribution of the pIgR contributes to the phenomena known as the Common Mucosal Immune System (22). The Common Mucosal Immune system argues that the gut can be protected by immune responses including secretory antibodies raised in the respiratory tract because of the widespread mucosal distribution of addressins that help traffic immune cells raised at one mucosal site back to other mucosal sites that express the addressin receptor; the pIgR is equally well distributed and facilitates antibody secretion at multiple mucosal surfaces, almost simultaneously. Local levels of pIgR expression can be adjusted since the pIgR expression locus is under the control of pro-inflammatory cytokines or their regulators (23, 24) and hence inflammation of a specific mucosal surface might see local elevated levels of IgA secretion through increased expression of pIgR. There is some redundancy in the secretion of antibody though as pentameric IgM carrying the J-chain is also secreted through the pIgR. The secreted IgA is intrinsically more resistant to mucosal proteases (25), most likely because cleavage sites are masked by the present of ‘secretory component’ (26, 27), part of the cleaved pIgR that is liberated during apical trafficking of the immune complexes, which stays attached to the antibody following passage through the epithelium (28, 29). Secretory component may be important in the gut where it interacts with gut mucins and may anchor secretory antibody in the lumen (30).

Our group was particularly interested in the role of sIgA in infection, especially bacterial infection, and developed a pIgR knockout mouse (pIgR^-/-^) in the C57BL/6 (BL/6) background, in collaboration with Per Brandtzaeg, a significant figure in the discipline of mucosal immunology, and Finn-Eirik Johansen from LIIPAT in Norway (12). Our research was led by a talented graduate student, Tania Uren and it was felt that the compensations seen in the IgA-deficient mouse, i.e. increased secretion of IgM (31), would be absent from the pIgR^-/-^. Uren and her colleagues showed that this pIgR^-/-^ mouse had neither sIgA nor secretory IgM in its secretions but had compensatory increases of IgA in the serum. Importantly, the inflammation first noted by Johansen in his pIgR knockout mouse made on a B129 genetic background (32), was recapitulated in the C57BL/5 mice. Uren also documented that deletion of the pIgR led to a 3-fold increase in the number of sub-epithelial B cells in the pIgR^-/-^ mice (12).

Our BL/6 pIgR^-/-^ mouse was developed to study the role of sIgA in infection immunity, particularly to the Gram negative enteric pathogen *Salmonella enterica* var Typhimurium, which is subject to growth control in the B129 mouse by Nramp1. Following the textbook logic, we anticipated that *immune* BL/6 pIgR^-/-^ mice would show increased epithelial invasion by *S.* Typhimurium compared with normal mice. Instead, we found that, while immune mice produced high levels of secretory IgA specific for *S.* Typhimurium, and that this antibody was present in the gut, the bacteria invaded into the mouse epithelia at similar rate to that observed in naïve mice. While there was considerable variability, the levels of invasion into *naïve* C57BL6 mice were generally lower than for naïve or immune pIgR^-/-^ animals (33), arguing a role for protection of the epithelial surface by ‘innate’ sIgA, possibly secreted by B-1 cells as polyreactive antibody.

This phenomenon was further explored by Wijburg and colleagues (34). She used naïve pIgR^-/-^ and normal mice, that were co-housed with a normal or pIgR^-/-^ *S.* Typhimurium-infected ‘shedder’ animals to determine whether innate sIgA could afford any protection in a more natural challenge model. In short, the studies showed that pIgR^-/-^ naïve mice i.e. lacking secretory antibodies were at significantly greater risk of infection than naïve BL/6 naïve mice. More recent data suggests that if mice are provided with a 10-40µg bolus oral doses of *Salmonella*-specific IgA monoclonal antibody they may show reduced infection into the epithelium infection (35), however the protective window was measured in minutes, and sufficient fully virulent bacteria typically translocate through the epithelium to establish a lethal infection in this protection model.

## Is specific sIgA infection blocking?

The adaptive immune response produces antigen-specific serum antibody detectable against the key outer membrane antigen of *S.* Typhimurium, lipopolysaccharide (LPS), which is detectable by one week (secretory IgM) or two weeks (sIgA) after oral infection with attenuated *S.* Typhimurium (33). In this model, this antibody level increases until 3-4 weeks post infection with the attenuated strain BRD509, and persists for more than 9 weeks. If the role of this antibody was to stop infection by the pathogen, then the kinetics of production are too slow to inhibit passage of the pathogen through the epithelium, which occurs within hours of arrival in the gut (33), and which is very well established by 3 days (36). This will likely be the case for most acute infections, i.e. that the appearance of high affinity B-2 B-cell derived specific secretory antibody induced by infection, which may be capable of blocking infection, occurs well after the pathogen has arrived and entered the host, and sometimes been cleared from the host. If the kinetics of the specific sIgA antibody response are not aligned with naïve infection immunity, might secreted antigen-specific antibodies still serve another role in either limiting infection, infection-related pathology, and/or transmission?

It is expected that, from a patency perspective, the mucosal lumen is largely inaccessible to serum antibodies in the absence of damaged epithelial integrity, i.e. where plasma might be transudated. There is good evidence that specific sIgA is important in resolving some distinct infection-related pathologies that are driven from the luminal side of the mucosae. For example, in a model of toxigenic lumen-restricted bacterial infection (caused by Enterotoxigenic *E. coli* or *Vibrio cholerae*), successful oral vaccination was only observed where mice carried a functional secretory antibody system, and effective immunization was not seen in animals lacking pIgR (33). IgA antibodies have been shown to block binding of bacterial adhesion to the enterocyte surface (37), though some of this may result from changed physicochemical properties of antibody binding to the fimbriae (38), rather by the blocking of specific receptor-ligand interactions (39). Through coating with sIgA, the antibodies may serve a role in inhibiting transmission of egressing viruses such as HIV (40) and SARS-CoV-2 (41).

There are many studies that demonstrate that specific secretory IgA is induced by infection, especially mucosal infection, and some studies using monoclonal antibodies that demonstrate both pathogen adhesion resistance, and intracellular neutralization of viral pathogens including rotavirus and influenza virus A (42, 43). In intracellular neutralization, the pIgR carrying sIgA is trafficked towards the apical surface of the epithelial cells and these trafficking endosomes fuse with endosomes carrying inbound virus, i.e. coming from the apical surface of the infected mucosal epithelium. Studies by Greenberg and colleagues demonstrated that this neutralization could occur even when the specificities of the IgA antibodies were non-neutralizing *in vitro*, arguing that conserved viral replication intermediates were available as intracellular neutralizing sIgA targets. How important intracellular neutralization is versus serum antibody-based neutralization is, as yet, unclear, but humans with IgA deficiency are at increased risk of rhinosinusitis (44) though, more often, IgA deficiency is associated with autoimmune disease and allergy, including coeliac disease (45–47), though again the effects can be subtle. There are some other even more cryptic infection-related effects seen when the secretary antibody system is disabled by loss of the pIgR or exploited for other reasons. For example, mice lacking the pIgR are more susceptible to *Mycobacterium bovis* strain BCG than normal mice (48) in an infection model considered to be largely CD4+ T cell-dependent (49). *Streptococcus pneumoniae*, which selectively binds to the pIgR through the bacterial surface protein CpbA, is trafficked across epithelial cells in culture by the pIgR (50, 51). Given that the pIgR is predominantly expressed on the basolateral surface of epithelial cells, is this a means by which the pathogen is transported to the mucosal surface?

Overall though, there is a clear argument that, unless the infection is chronic, there is little opportunity for specific sIgA to block epithelial invaision by pathogens because of delayed kinetics, though sIgA may interrupt subsequent transmission through coating of viruses and bacteria. Studies in naive mice lacking the pIgR suggest that polyspecific sIgA may afford some protection against infection, but the efficiency of many pathogenic viral and bacterial ‘invasion’ systems would suggest that this polyspecific antibody is insufficient to stop overt pathogen entry.

## IgA in antigen excretion

If we reconsider sIgA’s role to be predominantly antigen excretion, rather than infection blocking, we can explore its potential for removing pathological antigens from the systemic compartment, and the potential specific sIgA may have in treating chronic inflammatory diseases of the mucosae. For example, can specific sIgA be used to alleviate allergy or autoimmunity, through reducing the antigen burden in the epithelium *via* active antigen excretion? Such targeting might include providing low oral or intranasal doses of allergen(s) in a formulation that will induce IgA. Allergy is reportedly common in those with natural IgA deficiency; as recently reviewed by Cinicola et al. (52) and control over diseases including asthma, rhinitis and dermatitis might be achieved through targeted antigenic excretion. It is therefore helpful to look at the pathologies present in the pIgR mouse and where the inflammatory triggers may come from.

Our studies have shown that the IgG antibodies specific to individual members of the autologous microbiome are elevated in pIgR^-/-^ mice suggesting the failure to ‘excrete’ antigen drives a heightened humoral response against antigens from the microbiome (53). The same studies showed that untreated sIgA-deficient animals harbored 10-fold more culturable bacteria in their mesenteric lymph nodes, than wild type C57BL/6 mice. If these bacteria or their antigens are responsible for mucosal inflammatory conditions, then generating a specific sIgA response, or providing therapeutic antibodies with the same function, might reduce inflammatory conditions such as inflammatory bowel disease.

The observation that fecal microbiota is coated with IgA (54, 55) suggests that the gut microbes at least are in contact with polyreactive or specific antibodies and that, in the absence of sIgA, there could be a dysbiosis. In attempting to show this, we examined the ileal microbiomes of normal and pIgR^-/-^ animals. This study was done before there was general access to whole genome sequencing, and focused on the ileum where there is large natural variation in the microbiota, but the methods used suggested that the inherent variability between individual co-housed genetically identical mice precluded the definition of any specific changes due to the lack of sIgA (56). Others have found that humans with selective IgA deficiency had a clear dysbiosis (57) that might cause changes to antigen or microbial entry into the epithelium. Though less likely, the loss of IgA may result in a dysbiosis where less inflammatory microbiota are replaced by more inflammatory organisms.

More recent studies in mice had added some interesting details to the potential roles sIgA may have on the members of the interacting microbiome. Studies by Rollenske et al. demonstrated that coating with secreted IgA may protect the microbiome from environmental toxicity e.g. from bile, and from killing by bacteriophage (58), which would help shape the community. Notwithstanding our findings that immune mice lacking specific sIgA antibodies were generally as resistant to *Salmonella* Typhimurium as animals with intact secretion of IgA (33), except under ‘natural’ challenge (34), studies have revealed that, at very high bacterial cell densities, the presence of specific IgA can aid in the ‘clumping’ of *S.* Typhimurium, slowing bacterial division and enhancing clearance from the GI tract (59). The same studies showed that where bacterial taxa were unrelated, the presence of sIgA and consequent clumping could reduce conjugal transfer of drug resistance to sensitive populations. Of course, where sensitive members of the same (antigenic) species are ‘clumped’ with resistant members, conjugal transfer might equally be enhanced.

We have shown that congenital sIgA deficiency triggers a ‘failure to thrive’ syndrome in mice (60); we then further investigated the inflammation that was originally observed by Finn-Eirik Johansen and colleagues in their pIgR^-/-^ mouse that was produced in a B109 background (32). The gross inflammation we observed was slow to develop and, in older mice, was seen in about 1/2 to 1/3 of the animals by simple histology (60). Flow cytometric characterization showed that the lymphocytosis was much more frequent than it appeared in histological sections, and that elevated numbers of CD4+ and CD8+ intraepithelial lymphocytes (IELs) were present in the mice lacking secretory antibodies. These lymphocytes express α/β TCRs, not γ/δ TCRs, are CD69+ and, increasingly with age, express CD103 suggesting the cells are tissue resident. CD103 is a tissue homing integrin (αEβ7) (61) for the epithelial counter receptor E-cadherin and is a marker of active immune responses, at least in cancers PMID (62). What drives this lymphocytosis?

It is very tempting to speculate that the chronic peri-mucosal inflammation in the pIgR knockout mouse arises because microbiome-related PAMPs such as LPS and CpG DNA are increased in the sub-epithelium and that B cells accumulate in the pIgR^-/-^ mouse epithelium because of microbiome-associated antigens (33). In a ‘chicken-egg’ scenario, the inflammation may in turn drive an elevated epithelial permeability (60), leading to further entry by the microbiome, microbiome antigens and/or microbiome PAMPs. The enhanced permeability was first documented by Johansen (32) who saw increased albumin in the saliva and faeces; it follows that if albumin can leak through the epithelial barrier than microbiome-associated antigens can likely also cross the basement membrane into the tissues.

## A wider role for IgA in immune regulation

Unlike the short-lived effector T cell response, which can be quickly reduced by apoptosis (63–65), and very local T cell cytokine effects, the relatively long half lives of effector serum antibodies like IgG requires a countermeasure that can reduce their potency against residual antigen, and it is possible IgA fills this role. IgA has a shorter half life meaning that plasma levels reflect production levels and the moderation of activity can be brought through regulating B-cell numbers. Specific IgA will compete with other antibody isotypes for antigen in plasma. If specific IgA is able to outcompete IgG for antigen binding then the pro-inflammatory effects of complement activation are reduced, as demonstrated in studies of *Neisseria meningitidis*, where binding of IgA blocked access by complement-binding isotypes (66). Complement-activation brings inflammation through the generation of cleavage components like C5a and C3a (67). This competition by IgA with more inflammatory antibodies may not be the only mechanism through which monomeric IgA mediates an immunoregulatory effect.

Monomeric i.e. plasma IgA can bind to the FcαR1 expressed on myeloid cells including neutrophils and expression of FcαR1 is subject to inflammatory cytokines such as IL-8, TNF-α, and N-FMPL (N-formyl-methionyl-leucyl-phenylalanine) a chemotactic mimic of the formylated n-terminus of many bacterial proteins (PMID (68). Human FcαR1, also known as CD89, has multiple splice variants, some of which will likely have different functional features because they lack key domains (69). The FcαR1 is incapable of signalling unless it complexes with the FcRy (70) and Jacob et al. referred to FcαR1 as the ‘silent housekeeper’ (71). The engagement of monomeric IgA with FcαR1 has some unexpected consequences. Firstly, there is partial phosphorylation of the ITAM region of FcRγ-associated FcαRI, an immunoregulatory phenomenon mediated through SHP-1 tyrosine phosphatase, the recruitment of which leads to deactivation of other immunoreceptors, preventing inflammatory processes. The inhibition of especially neutrophil function by engagement by antigen specific, monomeric IgA could be an important step in reducing inflammation.

## Conclusion

**Figure 1:**
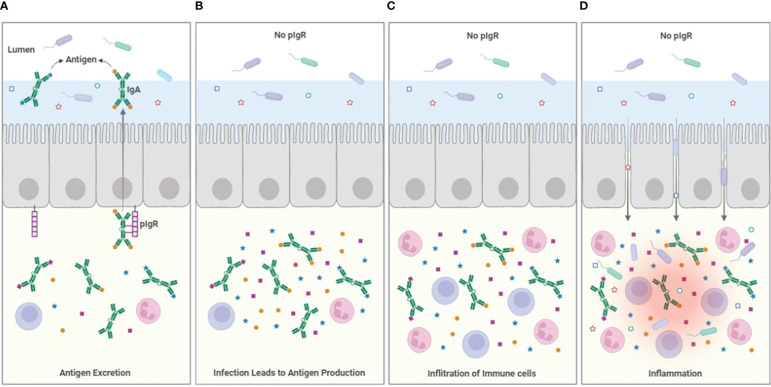
Model for antigen excretion. **(A)** The pIgR serves to excrete antigen bound to dimeric IgA, reducing the sub-epithelial antigen burden. Antigen excretion can occur through polyspecific antibody from B-1 B-cells, but the efficiency of the excretion process is improved by the induction of specific IgA that occurs during e.g. infection. **(B)** In the absence of pIgR (or the secretable antibodies dIgA and pIgM), antigen accumulates in the sub-epithelial tissues. **(C)** The accumulation of infection- and microbiome-related PAMPs and antigen drives the infiltration of immune cells, increasing inflammation. **(D)** The cytokines produced by the inflammatory cells weakens the integrity of the epithelial barrier allowing ingress of bacteria from the lumen into the tissues, increasing inflammation further. The loss of epithelial patency also results in leakage of serum proteins including albumin into the lumen.

The immune system is characterized by complementary features that extend from cell biology with pro- and anti-apoptotic pathways, to cellular functions where e.g. regulatory T cells attempt to restore homeostasis after aggressive activity by effector T cells. It should be expected that the humoral immune system has similar regulatory features. The potency of the long half-life antibodies like IgG which is mediated through high affinity binding and Fc-mediated effector functions will need to be moderated to maintain a balance and even the deletion of IgG producing B cells, though potentially important, will not impact circulating levels of antibodies for days to weeks. IgA has been seen as the antibody generated to protect mucosal surfaces. It could be posited that it does so, by using the secretory system based on pIgR to export antigen from areas where engagement with T cells and complement-fixing antibodies can lead to dangerous pathologies, rather than by preventing pathogens from binding and entering the epithelium (**Figure 1**). The IgA that is not excreted may also have important regulatory functions by competing with more inflammatory effector antibodies and through binding FcαR1, which can serve to limit myeloid cell activation. The full exploitation of IgA in chronic inflammatory and allergic diseases will require further investigation but the lessons learned from attempts to develop mucosal vaccines will be invaluable.

## Author contributions

RAS wrote and edited this manuscript and approved it for publication.
